# Beyond expectations: exploring the multifaceted role of positive gossip in organizations

**DOI:** 10.3389/fpsyg.2026.1824097

**Published:** 2026-09-18

**Authors:** Mustafa Doruk Mutlu, Aynur Çiğdem Ata, Eşe Yıldırım

**Affiliations:** Department of Business Administration, Yozgat Bozok University, Yozgat, Türkiye

**Keywords:** gossip, organizational dynamics, positive workplace gossip, public and academic organizations, qualitative research, SDG 8: decent work and economic growth

## Abstract

**Introduction:**

Gossip is a fundamental aspect of human communication that facilitates the expression of attitudes, beliefs, and opinions related to emotions. Within organizational settings, gossip frequently pertains to third parties who are not physically present and can be classified as either positive or negative based on its valence. This study focuses on positive gossip, specifically how it is perceived and experienced by employees as an organizational phenomenon.

**Methods:**

The study employed a phenomenological approach, with data collected through in-depth interviews conducted in two distinct organizational contexts in Türkiye.

**Results:**

The findings reveal that positive gossip is a significant phenomenon that can shape organizational dynamics, contributing to motivation, enhanced communication, and a positive workplace climate. However, the results also indicate that when exaggerated, positive gossip may lead to unintended negative outcomes, such as jealousy, perceptions of favoritism, and even potential misuse of power by those being praised.

**Discussion:**

This study contributes to the extant literature on organizational communication by offering a comprehensive analysis of the origins, content, circulation patterns, expectations of privacy, and dual impact of positive gossip on individuals and organizations. These insights underscore the necessity for a nuanced understanding of positive gossip, which is complex and multifaceted in nature.

## 1 Introduction

Organizational gossip is an informal form of communication among employees that involves positive or negative comments about a third party in their absence (Kniffin and Wilson, 2010; Spoelma and Hetrick, 2021). The present study focuses on positive gossip, which is a relatively underexplored phenomenon compared to negative gossip (Ugwu et al., 2022). In this study, positive gossip is defined as conversations about a third party who is not present that have a positive tone. From this perspective, the concept of “gossip” is retained, as the phenomenon examined involves conversations that include positive content about an absent third party, which is consistent with the established definition of gossip. It is imperative to emphasize that the term “positive” refers to the valence of the content of the conversation. In this context, it is not assumed that positive gossip is necessarily socially beneficial in terms of function or outcome. Therefore, positive gossip should not be equated with prosocial information sharing. In fact, research findings indicate that positively valenced gossip can also be experienced as being strategic, ambiguous, or even harmful.

Extant studies have yielded a variety of insights into the phenomenon of positive gossip from the perspectives of social networks, behavioral aspects, or the relationships among relevant variables (e.g., Grosser et al., 2010; Ellwardt et al., 2012; Kuo et al., 2015; Tassiello et al., 2018). The present research does not treat lived meanings as separate from behavior; rather, it focuses on the experiences of positively valenced gossip in an organizational context. It concentrates exclusively on positively valenced gossip, as its objective is to examine how employees encounter and interpret gossip with positive content. This focus diverges from the examination of positive gossip that also fulfills a prosocial role in terms of function. Despite the prevalence of qualitative research on workplace gossip in the extant literature, studies examining how employees experience and interpret positive gossip are relatively scarce. In this regard, the study’s specific focus on gossip that is positive purely in terms of content is important as it provides an opportunity to explore employees’ multifaceted experiences.

The national context of this study is Türkiye. This national context is significant for the study because social networks can shape the content, function, and perception of gossip (Khalid et al., 2025; Kruidhof et al., 2026; Küçük et al., 2025). In this study, culture is not treated as deterministic; rather, it is viewed as an interpretive framework that helps understand how positive gossip, occurring within two distinct organizational contexts, is experienced and interpreted by the actors involved. This perspective allows us to explore how general cultural trends in Türkiye translate into unique practices within public and academic spheres characterized by different organizational logics, and which values actors prioritize in the course of this process.

In contrast to extant studies, which characteristically draw parallels between positive and negative gossip, this study aims to address the gap by employing a phenomenological analysis in both public and academic contexts. This approach is intended to facilitate a more profound comprehension of the underlying dynamics that underpin positive gossip. By doing so, it aims to uncover the context-specific dynamics of positive gossip, its initiation, dissemination, and effects on individuals and organizations. This study enhances our understanding of organizational gossip by distinguishing positively valenced gossip from gossip that is assumed to be positive in both content and function. Additionally, it reveals how cultural codes in the Turkish organizational context, such as trust, relational closeness, motivation, and informal communication, shape the interpretation of positive gossip. Thus, it shows that positive content does not predetermine positive function; in fact, it reveals that positive gossip emerges as an ambivalent relational phenomenon. On the one hand, it can foster perceptions of appreciation, closeness, and motivation, whilst on the other, it can serve as a strategic tool or generate negative perceptions rooted in jealousy, privacy concerns, and favoritism.

Grounded in this theoretical framework, the study addresses the following research questions: Under what circumstances is positive gossip initiated in public and academic organizational settings? How do employees experience and interpret the content, motives, boundaries, and effects of positive gossip in these settings?

By addressing these questions, this research provides new insights into the ways in which organizational settings shape the dynamics and outcomes of positive gossip. The demonstration that positive talk can be perceived as appreciative and socially beneficial in some situations, yet as strategic, intrusive, or negative in others, provides a more nuanced explanation of positive gossip. By offering this multifaceted perspective, the study contributes to the extant body of knowledge concerning organizational communication, providing a nuanced understanding of positive gossip as a context-dependent phenomenon.

## 2 Theoretical background

This section will evaluate the literature concerning the concept of gossip and its functions. The theories about positive and negative gossip in organizations are then presented. Finally, the chapter concludes by explaining positive gossip in detail.

### 2.1 The concept of gossip

Gossip, a phenomenon as old as human history, can be encountered in every culture and environment, making it very difficult to avoid (Baumeister et al., 2004; Abbajay, 2014). Gossip is the act of talking about others when they are not present (Peters et al., 2017). Research indicates that nearly two-thirds of all conversations between two people are related to third parties (Emler, 1994). Further, in an organizational context, research has revealed that 90% of employees engage in gossip. They typically discuss their superiors’ behavior, alleged relationships between colleagues, and whether colleagues can adequately contribute to tasks (Dores Cruz et al., 2021).

It is a commonly held belief that gossip is frequently associated with negative, provocative, and embarrassing thoughts concerning the individual at whom it is directed. However, it is important to note that gossip may involve positive, negative, or neutral evaluations of absent others and is not necessarily intended to be malicious or defamatory (Abbajay, 2014). Ben-Ze'ev (1994) posits that there is an equal distribution between negative and positive information in gossip exchanges, an assertion that challenges the prevailing notion that gossip is predominantly concerned with humiliation or defamation. Recent research (Dores Cruz et al., 2021) has also demonstrated that individuals disseminate positive, negative, and neutral information about others.

Gossip has been viewed as a mechanism through which social groups share evaluative information and convey social and cultural meanings (Dunbar, 2004) and has also been demonstrated to facilitate the transmission of social and cultural dynamics (Baumeister et al., 2004). This phenomenon has been observed to transpire as a spontaneous, informal, and social transmission activity (Noon and Delbridge, 1993; Michelson and Mouly, 2000). In this respect, gossip can influence social bonds and trust in different ways depending on context, content, and interpretation (Ellickson, 1991). In an organizational context, these relational functions can be critical because they can potentially influence how employees interpret the behavior, reputation, achievements, or social standing of their colleagues.

This study uses social exchange theory as an interpretive framework. From this perspective, gossip is a form of communication through which socially valuable resources, such as information, approval, appreciation, reputation, status, and relational closeness, are exchanged (Blau, 1964; Cropanzano and Mitchell, 2005; Rosnow, 2001; Martinescu et al., 2019). Within this theoretical framework, the interpretation of positive gossip becomes paramount. Discussions of this nature, that is, concerning individuals who are not present, have the capacity to disseminate sentiments of appreciation and support. Conversely, they have the potential to evoke emotions such as jealousy and antipathy, along with perceptions of insincerity. Accordingly, the subsequent section will direct its attention towards the phenomenon of positive gossip as an ambivalent organizational phenomenon.

### 2.2 Positive gossip in organizations

Gossip can be seen as a nuisance to the proper functioning of organizations (Grosser et al., 2010). In particular, negative gossip can often lead to wasted time, hinder productivity, and as undermining employee morale while draining their energy (Michelson and Mouly, 2004; Wu et al., 2018; Ye et al., 2019).

However, the importance of recognizing that this phenomenon is a recurrent part of communication and interaction within an organization should be emphasized (Noon and Delbridge, 1993). Furthermore, Michelson and Mouly (2004) have argued that, although gossip is generally viewed as negative and avoidable, it can have both intended and unintended benefits. Gossip is a repeating form of communication within organizations and can serve relational and informational functions, such as conveying group norms, maintaining relationships, and helping managers and employees gather information about their surroundings (Rosnow, 1977; Grosser et al., 2012). Thus, gossip may provide insight into organizational culture, rules, and boundaries (Baumeister et al., 2004; Grosser et al., 2010). However, these potential functions do not imply that gossip should be endorsed as a desirable practice. Rather, they demonstrate why gossip persists in organizational life and how its outcomes are dependent on content, intent, relational context, and interpretation. From a social comparison perspective, gossip can shape how employees evaluate themselves in relation to others. However, these comparisons can lead to reflective or problematic outcomes depending on how they are viewed (Michelson and Mouly, 2004; Noon and Delbridge, 1993; Wert and Salovey, 2004).

This study focuses on a specific form of organizational gossip that carries positive valence. In this sense, positive gossip can include conversations with positive content such as the third person’s behavior, competencies, and achievements (Ellwardt et al., 2012). In the context of this study, the term “positive” refers to the valence of gossip. Thus, it does not inherently guarantee that its functions will also be prosocial or harmless. This definition distinguishes the positively valenced type of gossip at the center of this research from prosocial gossip, which is defined by its social utility function. Prosocial gossip serves a social utility function within an organizational context, such as protecting others, preventing harm, or fostering cooperation. From this perspective, unlike positively valenced gossip, prosocial gossip can have a positive function but also negative content. The current research focuses on positively valenced workplace gossip, examining how positive gossip is experienced and interpreted within an organizational context, regardless of its function.

Studies examining the phenomenon of positive gossip in an organizational context have generally focused on comparing it with negative gossip or identifying its positive relationships. Research in organizational contexts has found a number of favorable effects of positive gossip: work engagement (Ugwu et al., 2022; Shan et al., 2024), job satisfaction (Wang et al., 2022), innovative behavior of employees (Dai et al., 2022), cooperation and interpersonal trust (Sommerfeld et al., 2008), easier socialization of newcomers (Zong et al., 2021), reduced employee silence (Liao et al., 2022), reduced social shirking (Spoelma and Hetrick, 2021), reduced job insecurity (Jiang et al., 2019), and reduced individual stress (McAndrew et al., 2007). Recent studies that have focused on the interaction between managers and employees have found that positive gossip about employees by managers can mitigate the negative effects of patient abuse on nurses (Zoromba et al., 2025), for instance. However, a significant proportion of these findings are derived from quantitative studies encompassing the correlations, network models, and outcomes of positive gossip. This underscores the inherent limitations in attaining profound insights into the role of positive gossip in the daily lives of organizational actors, and indeed the manner in which it is perceived. A comprehensive table summarizing the qualitative and quantitative method differences of previous studies, their key findings, and the gaps they address in the literature is presented in Appendix B of the Supplementary material.

In the context of Türkiye’s cultural dynamics, understanding and interpreting the communicative dynamics surrounding positive gossip within organizations is vital. Three cultural dimensions commonly associated with Turkish culture, namely collectivism, high-context communication, and medium-to-high power distance, can provide a valuable framework through which to understand positive gossip in organizational settings (Hofstede et al., 2010). First, in Türkiye, a collectivist society, relational ties, and interpersonal closeness are often prominent in organizational life, and communication practices such as gossip can be intertwined with relationship maintenance, trust, indirect expression, and boundary management. Various studies have found that, from a social exchange perspective, gossip may have certain roles beyond mere information sharing in such contexts (Martinescu et al., 2019). Second, high-context communication may play a significant role in the more widespread use of gossip within the scope of indirect and implicit forms of expression. Third, the strong hierarchy and long-term employment ties within public organizations in Türkiye, which is the focus of this study, can influence communication dynamics and facilitate social exchange among employees. Furthermore, these contextual dynamics can shape the interpretation of evaluations of third parties who are not present. Previous studies in similar organizational contexts in Türkiye have shown that communication types such as gossip influence employees’ experiences and social interactions (Küçük et al., 2025).

The present study attempts to understand the phenomenon from the participants’ perspectives using a qualitative design in two separate research contexts. It examines various aspects of positive gossip, including how and why it is initiated, the topics it covers, its causes, confidentiality, and its effects from employees’ perspectives. Furthermore, drawing on the principles of social exchange theory, this study investigates the reciprocal nature of positive gossip-focused interactions in shaping social exchange. In doing so, rather than examining positive gossip in light of existing assumptions that consistently portray it in a positive light, the study approaches the phenomenon with a critical and nuanced perspective. To this end, with its distinct methodological approach, scope, and context, this research contributes to the development of a deep, comparative, and comprehensive understanding of the role of positive gossip in organizations and its dual outcomes.

## 3 Research design and method

The present study adopts a social constructivist ontological perspective, positing that the organizational reality is a construct of its participants, shaped by their interactions and reflections. Consequently, this study is grounded in individuals’ subjective experiences and their perception of the social world. The epistemology of this study is interpretivism, in line with the social constructivist approach. The study’s interpretivist approach focuses on participants’ subjective experiences of ‘positive gossip’ in their work contexts to understand the social world from their perspectives (Burrell and Morgan, 2019).

In accordance with the philosophical orientation, the qualitative research method of phenomenology was selected for the examination of employees’ lived experiences regarding positive gossip in organizational settings. The aim of phenomenology is to shed light on the fundamental nature of a phenomenon by examining how individuals perceive and interpret their lived experiences (Merriam, 2009). In this method, the researcher defines participants’ experiences and explains “what” and “how” they experienced them (Creswell, 2013).

During the data collection phase, participants were asked questions about the settings and situations in which they experienced the phenomenon in question (Creswell, 2013). Data analysis was conducted through a phenomenological process involving the identification of themes that reflect the essence of the participants’ shared experiences (Van Manen, 2016). The present study aligns with the fundamental principles of phenomenology by emphasizing the subjective meanings and interpretations of positive gossip, thereby enabling a more nuanced understanding of the phenomenon within organizational contexts. In this study, Merriam’s (2009) approach to phenomenology was employed to ensure an in-depth examination of the phenomena under investigation. This included events, experiences, perceptions, orientations, concepts, and situations.

Previous studies have identified general trends, outcomes, and correlations related to gossip and have provided valuable insights into workplace gossip. For this reason, a qualitative approach was deemed appropriate due to its ability to capture the complexity and depth of the phenomenon under examination. However, the extant literature on this topic is somewhat lacking, as there is a paucity of empirical studies examining how employees define and interpret positive gossip within their daily organizational contexts. This lacuna in research suggests a potential opportunity for the qualitative approach employed in this study to offer valuable insights. Given the complexity and context-dependence of positive gossip, this study presents an examination of employees’ narratives regarding positive gossip in two organizational contexts using a qualitative approach. The phenomenological methodology employed in this study addresses this gap by emphasizing the lived experiences of employees in public and academic institutions. This approach allows for the identification of context-specific variables and nuanced interpersonal dynamics that might be otherwise obscured by quantitative analyses (Patton, 2002). To illustrate this point, the complex dynamics of positive gossip and its effects on individuals and organizations have been examined.

The methodological choice to use in-depth interviews to directly obtain detailed information on the causes, forms, and perceived consequences of positive gossip from the perspectives of those involved provides the study with two advantages. First, it complements existing quantitative findings by enabling a deeper understanding of the phenomenon within an organizational context. Second, it has made it possible to address positive gossip as a multifaceted organizational phenomenon from a more holistic perspective.

Qualitative research focuses on how individuals construct reality within their interactions with the social world. In this method, the researcher seeks to understand the meaning of a phenomenon and how people interpret their lives and experiences (Merriam, 2009). Patton (2002) defines the qualitative research method as an approach in which the researcher does not attempt to manipulate the phenomenon of interest but rather seeks to understand it within context-specific settings (cited in Golafshani, 2003, p. 600). According to Hoepfl (1997), unlike quantitative researchers who aim to determine causality, make predictions, and generalize findings, qualitative researchers aim to explain, understand, and draw inferences about similar situations (cited in Golafshani, 2003, p. 600). In this context, the present study seeks to understand how positive gossip is perceived and interpreted by employees in their organizational environments.

### 3.1 Participant selection and context

In qualitative research, the sample is purposefully selected to generate the rich and relevant information needed for in-depth exploration, rather than through random selection or statistical generalization (Glesne, 2011). In phenomenology, however, purposive sampling continues until a sufficient depth of understanding is achieved and ends when no new additional information can be obtained from the participants (Merriam, 2009). The participants in the present study were selected using the purposive sampling method and on a voluntary basis, with initial access provided through the authors’ professional and social networks. The criteria for participation were determined by considering the interaction and communication environment necessary for the perception and experience of gossip; specifically, participants were required to be currently employed at the relevant organization and to have worked there for a sufficiently long time to have been able to observe and experience informal communication practices in the workplace. Participants were selected from different hierarchical levels, departments, and organizational roles to ensure diversity in their perspectives.

The research sample consisted of two public institutions operating in two different contexts in Türkiye: an academic institution, and a public agency. Interviews were conducted with a total of 26 individuals from these institutions; 12 were employees of the public agency (eight men, four women) and 14 were academic staff (five men, nine women). Overall, the sample included 13 male and 13 female participants, constituting a balanced gender distribution. With respect to institutional background, the participants represented two distinct public sector contexts: one characterized by bureaucratic and administrative work, and the other by academic and performance-oriented work. This contextual diversity was important for understanding how positive gossip is experienced and interpreted in different organizational settings.

None of the participants refused to be included in the sample. To provide a clear overview of the participant profile, a table is presented in Appendix D of the Supplementary material. This table summarizes the participant characteristics systematically available in the study. Detailed demographic information, such as age, educational level, and tenure, was not systematically collected during the data collection process. Therefore, to avoid retrospective reconstruction and to preserve the accuracy of the participant profile, these characteristics were not reported in detail. To ensure data saturation, the adequacy of the sample was assessed based on the repetition of patterns in the interviews and the fact that additional interviews primarily served to elaborate on existing categories rather than introducing new thematic directions; at this point, the sample was deemed sufficient to meet the study’s objectives.

The decision to focus on public organizations in this study stems from the fact that public institutions in Türkiye generally operate within a context characterized by hierarchy, strong ties, and a more stable work environment; this stands in contrast to the dynamic and fast-paced work environment typically found in the private sector (Aboramadan et al., 2021). The intensity of hierarchy has been demonstrated to impede the effective functioning of formal communication channels or to prompt employees to favor informal communication, which involves subtle messages and implications over openly expressing their feelings (Wu et al., 2021). Additionally, employees in Türkiye, particularly in the public sector, enjoy good job security (OECD, 2023). This leads employees to form longer-term relational bonds; in turn, this can increase informal communication and gossip, particularly in unmanaged areas (Küçük et al., 2025). For these reasons, positive gossip, a form of communication, can be a critical factor in influencing social dynamics within an organization, especially in the public sector.

These two contexts were selected because they offer different organizational conditions for understanding and interpreting how positive gossip is experienced. By analyzing the comprehensive dataset collected from these two organizations, which operate in different contexts but belong to the same cultural sphere, it has become possible to further illuminate how positive gossip is perceived and interpreted. The public institution, characterized by hierarchical and bureaucratic norms, has the potential to provide insights into the content of positive gossip and how it functions within rigid organizational frameworks. By contrast, academic institutions, with their performance-oriented and competitive environments, offer a unique context for understanding the dynamics and effects of positive gossip. When these two contexts are considered together, it makes it possible to conduct a more nuanced examination of how positive gossip is perceived and interpreted by organizational actors in the public sector.

### 3.2 Data collection

To collect data, semi-structured interviews were conducted with 26 staff members from public and academic institutions, yielding a wide range of perspectives. All interviews were conducted face-to-face in Turkish between January and February 2022. The interviews generally lasted between 30 and 35 min. While longer interviews might have elicited additional details, their focused and semi-structured nature allowed participants to provide rich and relevant accounts of their experiences with positive gossip. The researchers did not interfere in the nature or progression of the interviews; rather, participants were encouraged to elaborate on their experiences when needed, which resulted in some interviews lasting nearly an hour. Additionally, with the participants’ knowledge and consent, the interviews were audio-recorded, and participants were assured that their responses would remain confidential.

Before the interviews began, participants were briefed on the conceptual framework underlying positive gossip in the context of this study. Participants were then requested to share their reflections and experiences concerning the dissemination of positive gossip regarding third parties who were not in attendance. The interview questions explored the causes, subject matter, relevant actors, conditions under which positive gossip arises, expectations of confidentiality, and the perceived effects on employees and organizations. The participants provided detailed descriptions of the initiation, timing, location, and confidentiality expectations of positive gossip. Furthermore, they shared their thoughts on the effects of positive gossip, including both its advantages and disadvantages. In conclusion, the participants’ assessments of positive gossip within the scope of the study encompassed only positive comments regarding the absent party in terms of content (e.g., success, skill, and performance). While acknowledging that reaching participants through the authors’ professional and social networks may have influenced participant selection, communication, and interpretation processes, it is also important to note that this method facilitated trust and openness in discussing informal communication practices. The interview questions employed in the data collection process for this study, along with a detailed list of the focus of each question, are provided in the supplementary material to ensure transparency (see Appendix A). The objective of this section is to elucidate the methodological explanations and findings presented in the main text (see the Supplementary material for details).

### 3.3 Data analysis

An inductive approach, developed based on participant narratives, was adopted in the coding and theme development process. Social exchange theory was not used in the identification of the codes and themes obtained from the research data; rather, it provided a theoretical framework for interpreting the main themes obtained within the scope of the research. Furthermore, the theory offered a comprehensive overview of the role of positive gossip in social exchange processes, including social approval, appreciation, reputation, relational closeness, and information, within the contexts examined.

The data were analyzed based on Colaizzi’s (1978) phenomenological method, particularly its emphasis on significant statements, the formulation and clustering of meanings, and thematic organization. In line with this approach, all transcribed interviews were read repeatedly to develop in-depth understanding of the participants’ contextual experiences and perspectives regarding positive gossip. The analysis focused on narratives referring to positively valenced comments about an absent third party, in line with the conceptual framework presented to the participants before the interviews.

Coding was performed using the NVivo software, thereby facilitating data organization and management. After multiple readings of the transcripts, key statements related to the concept of positive gossip, which was presented to the participants prior to the interviews, were identified and coded according to the study’s framework. Similar codes were grouped into broader categories, and these categories were then organized into overarching themes. Through this iterative process, four main themes emerged, forming the core structure of the study. Rather than imposing codes and categories in advance, an inductive approach was used, as they were derived from the participants’ narratives in both contexts. Although the interpretation was based on the participants’ narratives, it was approached within the framework of social exchange theory, which explains the dynamics of reciprocity and relational balance in an organizational context; the theory played a fundamental and enriching role during the phenomenological analysis phase. In this sense, social exchange theory was not used as a predefined coding template; rather, it was applied after the coding and categorization process to examine the emerging themes within broader relational dynamics. Since the interviews were conducted in Turkish, the quotations presented in the analysis text have been translated into English by the authors. To preserve meaning and contextual nuance to the greatest extent possible, the translations were reviewed by the co-authors. Particular care was taken to preserve the intended meanings of the participants in the English quotations. Therefore, the translated excerpts have retained some informal, fragmented, or indirect expressions in order to reflect the participants’ original speech patterns as closely as possible.

The aim of this study is to understand the phenomenon of positive gossip using qualitative methods and to meet high-quality standards. Accordingly, during the analysis phase, the analytical process, conducted meticulously within the theoretical framework, sought to establish meaningful consistency with the empirical findings (Tracy, 2010). Additionally, researcher triangulation was applied to ensure the reliability of the study. During the analysis phase, prolonged interaction and intensive description were also used to ensure the direct transcription of participants’ quotes (Lincoln and Guba, 1985).

The study also focused on ensuring intercoder reliability, and the process was conducted in accordance with Colaizzi’s (1978) well-known approach. Accordingly, after the initial coding by the third author, the second author recoded the data individually. During the categorization of codes, the two coders engaged in intensive, iterative interaction to reach consensus on the data and thereby establish the final themes. This approach strengthened the reliability of the final thematic structure and contributed to the validity of the research. To enhance transparency in the analytical process, a detailed table illustrating the relationship between key statements, codes, categories, and themes is presented in Appendix C of the Supplementary material.

## 4 Results

The findings can be interpreted in relation to three broad dimensions that are becoming increasingly prominent in the literature on workplace gossip: antecedents, relational dynamics/functions, and consequences (Sun et al., 2023). In this context, the theme “Contextual Content and Motives (Context-specific precursors and motivations)” addresses the contextual content of positive gossip, as well as the motivations and conditions surrounding its emergence. The theme “Social Boundaries and Conditional Privacy (Relational boundaries and interpersonal dynamics)” highlights the relational, interpersonal, and interactive boundaries within which such conversations circulate. The theme “Emergence and Circulation (Emergence Processes and Interactional Dynamics)” further clarifies how positive gossip emerges, develops, and progresses within organizational settings. Finally, the theme “Ambivalent Effects (Multi-level Consequences for Individuals and Organizations)” addresses the various consequences for both individuals and organizations.

The objective of this inquiry is to gain insights into the phenomenon of positive gossip in organizations from the participants’ perspectives. In this context, the research findings have been categorized according to four main themes consistent with an examination of the role of positive gossip in an organizational context. The thematic framework encompasses the context-sensitive content and interpretive motivations of positive gossip, its relational boundaries and conditional confidentiality, the environment in which it is initiated and disseminated, and its multifaceted effects on individuals and organizations. To clarify the description, labels were created for participants according to their organizational context. “P” refers to participants from a public institution, and “A” refers to participants from an academic institution.

### 4.1 Theme 1: contextual content and motives

Among the reasons why positive gossip is used as a means of communication is the sharing of information in the content of the gossip. While it was found that positive gossip was generally made about the professional behavior of colleagues at work in public institutions, it was understood that gossip in academic organizations focused more on performance-oriented academic success. Below are the findings obtained from the interviews held in public organizations. As can be seen, work, working life in general, and the way employees do their jobs are the subjects of such gossip.

P8: *It [positive gossip] can be about people’s personalities. In addition, work life and work-related issues are usually included in positive gossip.*

P10: *… it [positive gossip] is more about the way people do their job; that is, people are already revealing their characters while working, in fact, it is basically about the character of the person.*

The following statements show that positive gossip in academic institutions is predominantly related to academic work and performance.

A2: *We say* “look how well she gets academic incentives every year, how she works, she has publications”*, in other words how much effort she makes.*

A3: *We gossip [positively] about people’s achievements… if there is a success, for example,* “she/he made a project and won the first place or published so many articles” *…*

In the context of “Underlying Motives of positive gossip”, the inquiry into the rationale behind the provision of such positive speech and the interpretation thereof assumes significance. The participants’ experiences indicate that this phenomenon can emerge for various reasons, including the appreciation of a third party, the desire to share happiness, the implication of a vested interest, or the masking of jealousy.

P10: *There is this thing in our society about positive gossip: people perceive saying nice things about each other as a part of a long-term plan. He/she’s probably gossiping to look good for her. There may even be people who think that their kind words can reach that person. But if someone says something positive, I guess there might be something in it, there might be a closeness or there might be a relationship of interest with the person they are talking about.*

A2: *When I see the achievements of successful people, I like to talk about it everywhere because I feel like I have achieved it.*

A4: *I want the work to be seen and heard, that belongs to my friends. I wish the same could be done for me.*

One participant also pointed out that positive gossip can actually be used to mask negative thoughts and emotions.

A5: *As I said before, people gossip positively with malicious intentions… If we do not like a person and because it is not possible to say that he/she achieved such success at work and that he/she did not deserve to get it, the easiest way to show these feelings is to mask them. Through using round sentences like* ‘Did you see that he/she has applied [for an] associate professorship, I hope it will be good for him/her,’ *and pass over the subject.*

In Turkish social interactions, even words of praise may not be perceived as directly positive; they may be assessed in relation to trust, hidden motives or interests, and the social context in which they are spoken. Accordingly, it is understood that positive comments made about a third party who is not present may also be interpreted through indirect expressions, implied messages, sincerity or distrust, and relational positioning.

### 4.2 Theme 2: social boundaries and conditional privacy

In organizational life, positive gossip may sometimes entail a diminished expectation of confidentiality, particularly in contrast to negative gossip. However, as participant statements revealed, this does not imply that positive gossip is inherently shareable with all individuals. In fact, the participants indicated that positive gossip within the research context was voluntary among those who had closer relationships with each other. This finding contradicts the argument that positive gossip is easily shared among members of an organization (Grosser et al., 2010).

P10: *It happens among the* people *who come into contact with each other during the day.*

P12: *More like our close friends and the people you are trying to communicate with one-on-one.*

It is important to note that the participants found it appropriate to do this with people with whom they had mutual trust and sincerity. It was stated that some issues are avoided with people they do not feel close to. This suggests that, in the context of positive gossip, privacy and trust are conditional and, therefore, can vary according to the subject matter and the relationship between the parties.

P2: *So do not think only in terms of public institutions, in the same way [as the] private sector, a wrong word coming out of your mouth can cause misunderstandings. Whether it is positive or negative, the issue of trust is very important.*

A6: *In fact, this [positive gossip] happens where there is sincerity. For example, there is a certain group of sincerity both within the institution and in us, and it happens between that group. You do not gossip with any colleague outside of work, this (positive gossip) again happens with your most intimate group. In fact, [it is with] the people you feel closest to.*

The statements above suggest that positive gossip is still shared within certain relational boundaries. Trust and sincerity between the parties are necessary for positive gossip to circulate. Therefore, it is misleading to assume that positive gossip can circulate freely within an organization. The findings clearly show that positive gossip must be understood in the context of cultural dynamics, such as established closeness, sincerity, and the expectation of avoiding misunderstandings. For Turkish organizations, this pattern suggests that positive gossip is shaped by culturally significant factors such as sincerity, trust, and the careful establishment of interpersonal boundaries.

### 4.3 Theme 3: emergence and circulation

This theme examines the circumstances under which positive gossip is initiated within an organizational context. It considers the environment in which such gossip is most likely to occur and the circumstances under which it is most prevalent. The findings indicate that such gossip is often initiated in an unplanned and unintentional manner.

P12: N*o, it is not usually done consciously. You know, you open up spontaneously, finish the whole thing thoroughly.*

However, some of the participants stated that there may sometimes be a certain intention behind the initiation of positive gossip.

P10: *Well, if there is no personal expediency then it is spontaneous. But if he/she has an interest, he brings it there and makes a positive statement. I would say flattery.*

P9: *When the gossip is positive, sometimes it has a background… The gossiper usually comes with a request. Let us say her/his friend has a problem, and the gossiper wants to find a solution. She/he goes to the person who has power to offer a solution and puts [in] a good word about the friend in need… So, I think it’s more likely to come with a premeditated request rather than [being] spontaneous.*

With regard to the spatial and temporal parameters of positive gossiping within the organizational context, the participants indicated that this occurs predominantly in informal settings, mostly during periods of leisure.

A2: *… conversations [positive gossip] about the work environment seem to be more common at work. I do not know, we go to the cafeteria, and we talk [positive gossip] while eating.*

P3: *In the institution, it is usually done when people finish their work and go to sit for tea with each other, and usually by closing the doors… but sometimes it also happens that when you talk to people you come together with outside of work, it is also opened, so it is also mentioned there.…*

Furthermore, the participants indicated that positive gossip typically occurs in a convivial atmosphere within the organization. Conversely, an examination of the statements pertaining to the context in which these conversations take place reveals that they typically occur in person. Nevertheless, some participants in the academic organization assert that positive gossip can also occur online.

A9: *It’s also made on WhatsApp, but it’s usually done face-to-face.*

### 4.4 Theme 4: ambivalent effects

Within this overarching theme, the effects of positive gossip can be categorized into three distinct groups: positive, negative, and effects on the individuals involved in the gossip. Research in the field of organizational behavior suggests that positive gossip among employees can lead to strengthened bonds between colleagues (Ugwu et al., 2022). The participants in this study identified several positive effects of gossip, including its role in maintaining organizational cohesion, strengthening communication, and enhancing employee morale.

P10: *By the way, we work here with 20 people, 20 different characters, 20 different thoughts. If we perceive these people with their differences and then evaluate them with these differences, we cannot be united. We must surely find common ground. That’s why I think it [positive gossip] brings people together outside of these differences and keeps the institution together.*

P7: *It really makes people feel good and can change the perspective of those around them or show them what they do not see.*

The following statement emphasizes that talking about the problems experienced by friends strengthens organizational ties and creates positive effects on the sense of organizational belonging.

A6*: Well, I believe it strengthens the bonds. For example, I heard that my friend was thinking and worrying about my problem and talking about it when I wasn’t there. That’s when I feel more like I belong there. Well, in a friend group or at work… knowing that you are being thought of, knowing that you are well spoken [of] creates a positive effect at that moment.*

Some participants underlined that positive gossip strengthens communication within the organization and increases information sharing.

P10: *Positive gossip strengthens people’s communication.*

A3: *First, it [positive gossip] provides information in places [organizations] that are definitely more in number [of employees]. So, you have information about others.*

The effects of positive gossip on employees are generally positive. On the other hand, it was also found that talking about the achievements of other members through “gossip” emerged as a motivating factor among the participants interviewed.

A1: *It [positive gossip] happens among colleagues from time to time but mostly used by our rector at congresses or meetings in order to increase the success of the academics and to encourage them positively.*

P7: *The effect of positive gossip on our institution and employees is motivating. I mean, for one thing, it motivates me a lot.*

It was also observed that positive gossip can have negative effects in addition to the positive ones. It was highlighted that positive gossip can lead to negative feelings like jealousy and envy being directed towards the person being talked about.

A5: … *for example,* ‘Did you hear that so-and-so applied to be an associate professor, how nice, she/he will become an associate professor in a short time.’ *These discourses seem to be positive, but I think there is a little bit of jealousy or envy behind them*.

A10: *Unfortunately, when positive gossip is made among the colleagues, we have too many of them who want to abuse it or turn this current pleasant atmosphere in different directions with different and incomplete information. After a certain time, positive gossip begins to turn against that colleague (about whom positive gossip is made). Because, unfortunately, people cannot accept this because they cannot digest it.*

In addition, the interviewees stated that positive gossip can lead to negative gossip. They also underlined that positive gossip should not be exaggerated as this may have negative effects on people.

P10: *It disturbs if it is too much. If we say a good word or two about the friend who was just here, since it is in the nature of the conversation it is not very unusual, but if we praise him/her too much, I think there may be a reason underneath. I think there is definitely a relationship of interest or a relationship of love.*

A5: *…I think it has destructive effects in the organization when positive gossip takes place at the management level about the same person all the time, because none of us like it. While we are all in an environment, none of us likes to have one person be brought to the fore all the time like how beautiful that woman is, how nice and how well she dresses. We do not always like someone to be praised positively, as if good things have always been gathered in a person’s life and we do not have them. Because it reminds us of our own shortcomings, and we do not like to accept our imperfections as human beings. Instead of saying that I should work harder and be like them and be praised, I think it is possible to grab the other person’s leg and pull them down, this is done very often in my institution.*

This pattern is especially important in hierarchical organizational relationships. Repeated praise from organizational leaders can be perceived as more than just recognition. Conversely, it can be interpreted as symbolic promotion for the subjects of the gossip or as preferential treatment directed toward them. In this sense, positive gossip reveals how praise, comparison, and perceived justice interact within a given cultural and organizational context. It also shows that, particularly in hierarchical settings, excessive praise may not always be perceived as directly positive; instead, it can trigger suspicion, jealousy, or concerns about favoritism. In this sense, it highlights how positive gossip, praise, comparisons, and perceived fairness interact within a specific cultural and organizational context.

With regard to the effects of positive gossip on the person spoken about, research indicates that individuals tend to react negatively when they hear negative gossip about themselves (Wu et al., 2018; Ye et al., 2019). In this study, when participants were asked what they would think if they heard or knew about positive gossip about themselves, they stated that they would not complain because the content of the gossip was “positive.” In contrast, they may like it…

A4: *Honestly, I would be happy. Because as I said, I would like it if what I do is heard and seen…*

P11: *I mean, people like to feel that their friends and superiors see their effort in some way, we all like to be appreciated, which happens through positive gossip.*

A8: *It [positive gossip] affects me in a good way. It actually makes me positive… But on the other hand, I worry sometimes about having to make sacrifices as these people say nice things about me and I do not want to upset them.*

## 5 Discussion

This study explores employees’ experiences with and interpretations of positive gossip in public and academic organizational settings. The findings suggest that positive gossip should be understood in terms of being more than just its positive valence. It should also be considered in light of the conditions under which it arises, the relational purposes it may serve, and the outcomes it may produce. Positive gossip emerged as a common form of communication in both the organizations included in the study. While positive in terms of valence, positive gossip is not necessarily prosocial. In fact, the findings show that the effect of positive gossip varies according to the relationship, perceived intent, and context. It can range from fostering appreciation, motivation, and social bonding, to evoking perceptions of jealousy, suspicion, and favoritism.

In fact, the multifaceted findings obtained in the study can also be linked to the Turkish organizational context. The findings clearly show that participants did not interpret the phenomenon of positive gossip in line with its positive content. It is evident that, within the context of the study, participants considered whether the speaker was sincere, whether there was a hidden agenda, whether the comment might be misunderstood if repeated, and whether praise from a superior might signal favoritism. This suggests that the findings can be linked to culturally distinct interaction patterns such as relational closeness, indirect expression, trust, and sensitivity to hierarchy. Consequently, the Turkish organizational context does not merely function as a broad cultural label within the scope of this study, it also helps explain why positive gossip is interpreted as positive in some situations and as strategic, ambiguous, or unfair in others.

The findings of the research on the topics on which positive gossip can be made in an organizational context revealed that positive gossip can address a range of topics. Such topics include, but are not limited to, matters related to work performed, the personalities of colleagues, and the management style of the organization. They can also encompass the employees’ appearances, their talents, performance, and also matters unrelated to work. As can be seen, positive gossip in the work environment is not only limited to the issues within the workplace but can also address non-work-related issues. However, the emphasis placed on these topics varies across organizational settings.

Complete comparative analyses of the public and academic organization that served as sources of research data are beyond the scope of this study. However, some findings suggest context-sensitive patterns. In the public institution, positive gossip typically concerns professional conduct and colleagues, whereas in academia, it tends to focus on academic achievements. This differentiation may be due to the differentiation of the distinct culture of each institution. In other words, while the perception of a personal positive situation in the academic organization is measured by academic success, it is striking that a behavior-oriented evaluation would be made in the public institution. From this perspective, positive gossip can also signal which achievements, qualities, or skills are attracting attention in an organization. These findings suggest that the content of positive gossip can also provide information about corporate culture (Michelson et al., 2010).

Research findings have shown that positive gossip can occur both in organizational settings and in private life to acknowledge others’ successes and positive qualities. However, participants also emphasized that positive gossip may serve strategic or disguised purposes. From this perspective, positive gossip may sometimes be motivated solely by socialization. As Di Fonzo and Bordia (2007) suggest, the need to fulfill a sense of belonging, the desire to form social networks, or the desire to alter or maintain existing social networks can also lead to the emergence of gossip. However, beyond its apparent positive intentions, this study reveals more complex and less explored dimensions of positive gossip. Interpreting the findings within the cultural context in which the data were collected reveals that positive gossip is a form of communication encompassing a wide range of direct and indirect interactions. Participants’ statements imply that positive remarks about an absent person may also contain implicit comparisons, jealousy, or suspicion. This suggests that some actors go beyond using positive gossip solely for prosocial purposes. Positive gossip appears to serve as a socially acceptable way of indirectly expressing ambivalent or negative feelings, based on their experiences and interpretations. In fact, these emotions are sometimes subtly expressed through gestures or tone during positive gossip. By acting in this way, the individual can conceal true feelings while maintaining a socially acceptable facade. This particular use of positive gossip highlights an underexplored interpretive dimension in the literature on workplace gossip.

Furthermore, the study reveals that positive gossip can be strategically employed or interpreted to achieve various objectives within organizational settings. Participants’ accounts indicate that positive gossip can be read not only as a means of appreciation but also as a means of strengthening relationship ties and positioning for personal gain. It can be used to enhance an individual’s reputation or influence within an organization. Specifically, employees may seek to align themselves with successful colleagues or to influence third parties’ perceptions in a manner that aligns with their long-term objectives. This strategic use highlights the dual nature of positive gossip, which can serve both relational and self-serving purposes simultaneously.

Additionally, the results indicated that managers can also benefit from positive gossip by praising a third party to motivate others and to set a positive example. These contributing new insights emphasize the need to move beyond the perception of positive gossip as a purely harmless or constructive phenomenon, but rather as a multifaceted interaction of motives and outcomes. By doing so, they endeavor to align team behavior with organizational goals by strategically employing positive gossip. However, the findings also reveal that positive gossip in hierarchical relationships may not convey its intended meaning to employees. In particular, positive gossip repeatedly shared by management, which generally contains praise, can have unintended consequences. For example, it can go beyond strengthening the image of third parties who are not present and motivating other employees. In such situations, the perceived image of the third parties being gossiped about may, in fact, symbolically improve in the eyes of others. This can lead to the interpretation that management is treating this person preferentially or that they have formed an alliance with management behind the scenes. While managers strive to positively influence organizational dynamics from a motivational perspective, excessive, unfair, or unwarranted positive gossip can trigger negative emotions and perceptions, such as jealousy or the perception of favoritism, among other employees.

These findings underscore the distinction between positive-content gossip and gossip that is positive in both content and function. By emphasizing the interplay of various motivations and outcomes, the research reveals the multifaceted nature of positive gossip, contradicting the notion that it is entirely harmless and beneficial. In this regard, the study demonstrates that the valence and function are analytically distinct dimensions of positive gossip depending on actors’ experiences and interpretations within organizational contexts. This contribution necessitates a more nuanced understanding of the role of positive gossip in workplace contexts, particularly within both theoretical and practical discussions of organizational behavior.

When it comes to the question of among whom positive gossip usually takes place, another common finding in the institutions where the research was conducted was that although gossip was positive, it was mostly performed among close and trusted friends in both contexts. This finding contradicts Grosser et al.’s (2010) assertion that positive gossiping can be performed with anyone who works together in the same environment. In fact, in both contexts, it has been revealed that individuals are mostly selective about the person or people they communicate with, even while gossiping positively. These people appear as peer employees, non-managers, and same-sex fellows, especially the members of the organization they feel close to and trust. It can be said that the reason why the members of the organization behave selectively even when gossiping positively is that positive gossip mostly happens among people who want to strengthen communication and to whom continuity of friendship is an intended aim (Ugwu et al., 2022).

Despite the positive valence of the gossip, participants in both contexts within the Turkish public sector did not characterize this form of conversation as something that should be readily shared with others. Conversely, they emphasized the significance of interpersonal sincerity and trust as the prerequisites for such informal dialogues to unfold in a comfortable and meaningful manner. This finding indicates that these conditions influence the dissemination of positive gossip within the current cultural context. Consequently, the degree of camaraderie among employees does not necessarily translate into the function of positive gossip. However, it delineates the relational boundaries within which such content tends to circulate, irrespective of whether it is perceived as supportive, strategic, neutral, or carrying a masked negative implication.

As a result, gossip is one of the most important elements of organizational communication and the degree of confidentiality of this sharing stands out as an important issue. After all, it is not possible to expect every interaction that takes place informally in organizations to remain transparent (Dufresne and Offstein, 2008). A common finding from the interviews with the participants was that positive gossip involves, to some extent, lower expectations of confidentiality. However, the extent to which people expect confidentiality varies depending on the level of trust between those involved, the sensitivity of the topic, and the role of relational boundaries. From this point of view, the findings indicate that positive gossip differs from “confidential gossip,” which includes information that is asked to remain confidential (Fan et al., 2021).

It has been posited that the predominant rationale behind individuals’ engagement in positive gossip or their inclination to refrain from concealing their observations may be attributed to their perception that the subject of the gossip would derive motivation from being informed of it (Ugwu et al., 2022). However, the present study reveals a more nuanced reality: positive gossip affects individuals in diverse ways. While it may precipitate positive outcomes such as happiness or a sense of appreciation, it can also result in indifference or even negative feelings, such as discomfort or frustration. These findings challenge the prevailing assumption in the literature that positive gossip consistently yields favorable outcomes for the individual being discussed. On the contrary, this research uncovers that in certain circumstances, positive gossiping may result in undesirable negative consequences, including feelings of unwarranted jealousy and antipathy toward the individuals being gossiped about. Further, positive perceptions regarding these individuals’ performances can also lead to an increase in their workload, as their actions are perceived as exemplary. By highlighting these dual effects, this study contributes novel insights to the existing literature and calls for a reevaluation of the effects of positive gossip on third parties in organizational settings.

Organizational gossip occurs in unmanaged areas such as kitchens, canteens, corridors, or cafes where employees commonly gather during work breaks (Waddington, 2010; Mitra and Gilbert, 2012). Current research results are also in line with such observations, and have revealed that positive gossip occurs face to face and mostly in internal social meetings in both contexts. These unmanaged spaces provide a conducive environment for informal exchanges, including positive gossip, highlighting their role in fostering social cohesion, and communication among employees.

The extant research findings indicate that positive gossip is predominantly carried out in physical spaces, but it has also been found to occur on online platforms in academic contexts. This finding suggests that context may be a determining factor in the environment in which positive gossip occurs. From this perspective, especially in academic environments, the spread of digitalization and digital communication can lead to such informal interactions taking place in digital environments. Consequently, it is imperative to consider both digital media and traditional modes of communication when studying the dynamics of positive gossip within organizational settings. This finding suggests that positive conversations about work mostly occur within the boundaries of the workplace and in social meetings that occur in areas that are not managed during breaks. From this point of view, it can be posited that members of the organization at least tend not to undertake positive gossip outside the organization. Despite the preference for face-to-face communication, it has been posited that positive gossip can also transpire through online platforms within academic institutions.

Turning finally to the effects of positive gossip on the organization, research has shown that it can have some favorable influences on the organization, but it can also pose certain challenges. On the positive side, findings indicate that it can serve to reinforce positive behaviors within the institution, provide motivation for improving performance, and strengthen communication. These results, in fact, align with prior research demonstrating that positive gossip enhances cooperation and interpersonal trust among organizational members (Sommerfeld et al., 2008), reduces individual stress (McAndrew et al., 2007), and increases interpersonal convergence (Liao et al., 2022).

However, when taken to the extreme, it can also have negative effects such as jealousy, envy, feelings of competition, perceptions of favoritism, and the risk of turning into negative gossip. It has also been stated that the person who is constantly praised may begin to feel too powerful, and may abuse it. This pattern, where relationships and hierarchy shape interpretation, may be particularly relevant in Turkish work environments. In such contexts, repeated praise may be perceived as not only appreciation, but also a sign of special closeness, symbolic elevation, or preferential treatment. Therefore, excessive praise, especially when repeatedly directed at the same person, can trigger suspicions of hidden agendas and concerns about fairness. Based on these findings, although the positive effects of positive gossip are often emphasized in the literature (Liao et al., 2022; Spoelma and Hetrick, 2021), it can be concluded that attention should be paid to its diffusion and use within an organization, and that being positive in valence does not necessarily mean that it is absolutely harmless. This discovery also establishes a foundation for interpreting positive gossip through the lens of social exchange theory.

Although positive outcomes are frequently observed in the social exchange process, imbalances or negative perceptions may occasionally emerge when viewed from a social exchange perspective (Cropanzano and Mitchell, 2005). The results of the current study indicate that positive gossip can have multifaceted effects in an organizational context. In this context, the research findings suggest that positive gossip may circulate critical organizational resources, such as appreciation, approval, reputation, relational closeness, and information, informally. From this perspective, when actors engage in positive gossip, they do not merely convey the positive content within the message. At the same time, through such positive conversations, they can enhance the reputations of third parties not present in the setting, signal factors valued within the organization, and circulate symbolic resources that can strengthen relationships among those participating in the gossip. This, in turn, can influence dynamics related to motivation, commitment, power, and social bonds within the organization.

However, the research findings are significant in that they reveal these exchanges may not always be balanced or positive in nature. The research findings contribute to a social exchange understanding of positive gossip by demonstrating that it can also lead to strategic or negative exchanges. Indeed, the findings reveal that positive gossip can be strategically utilized by both employees and management. Employees can use positive gossip to mask their true feelings, pursue self-interest or flattery, or aim to influence a third party; management, on the other hand, can use positive gossip as a strategic motivational tool and indirectly signal aspects such as the characteristics employees should possess within the organization. On the other hand, when management is perceived to engage in positive gossip in a hierarchically biased manner or repeatedly targeting the same individual, it can generate negative emotions among employees, such as jealousy, distrust, a sense of favoritism, or exclusion. In conclusion, these multifaceted findings regarding positive gossip underscore the importance of maintaining balance in social exchange processes and highlight the potential consequences of imbalance.

### 5.1 Limitations and future studies

The present study was conducted over a 2-month period at two distinct institutions (academic and public) in the Turkish cultural context, which is clearly a limitation of the study. A further limitation is that certain participant demographics, such as age, level of education, and tenure, were not systematically documented. As such, the findings may not fully capture the dynamics of positive gossip in other organizational settings or cultural frameworks. Although they serve to help understand the phenomenon of positive gossip in depth, they may not fully capture how it operates in other sectors, such as private or other nonprofit organizations. Additionally, the study is qualitative in nature and thus is not designed to allow for the generalization of its findings to all organizations.

Given these limitations, future research avenues could broaden the scope of this study by examining the role of positive gossip in diverse contexts. Cross-cultural research would be particularly valuable in helping to understand how cultural norms shape the initiation, transmission, and reception of positive gossip. In addressing concerns regarding the generalizability of the results, increasing the sample size using quantitative methodologies could be investigated. Longitudinal studies are crucial to assessing the long-term impact of positive gossip on organizational outcomes. Finally, investigating the interplay between positive gossip and managerial practices could provide valuable insights into how leaders can strategically leverage positive gossip to enhance organizational performance.

## 6 Conclusion and implications

This study has explored how positive gossip is perceived by individuals in public and academic organizations. The findings of this qualitative study reveal that positive gossip, although often overshadowed by its negative counterpart in academic studies, plays a significant role in shaping organizational dynamics. However, it contradicts the notion, posited by prior research, that positive gossip is a rosy phenomenon that occurs in organizational contexts. Rather, the study’s findings indicate that positive gossip is a complex occurrence that, when examined in depth, can have both positive and negative consequences. For these reasons, positive gossip in organizations should be monitored carefully, with efforts made to benefit from its positive outcomes while minimizing its potential negative consequences.

The findings of this study have significant implications for theoretical frameworks and practical applications of positive gossip. From a theoretical perspective, the study contributes to the existing literature on positive gossip by offering a thorough analysis of its dynamics, contexts, and outcomes. Specifically, the research challenges the common, oversimplified notion that positive gossip is always constructive by revealing its dual nature (Ellwardt et al., 2012). The study also shows that, while positive gossip can circulate socially valuable resources, it can also be used excessively, strategically, or in a biased manner by managers or employees, which can potentially disrupt the balance of exchanges. These findings contribute to the existing body of knowledge in organizational communication and offer new perspectives for social exchange theory.

From a practical perspective, this study provides important insights for organizational managers seeking to create a positive work environment. By acknowledging the dual nature of positive gossip, leaders can strategically leverage it to motivate employees and strengthen social bonds. For example, by strategically using positive gossip, managers can develop a culture of appreciation and cooperation within the organization by acknowledging achievements and setting an example. In this context, managers can intentionally create opportunities to highlight individual or team successes during their interactions with employees. However, managers should be aware that positive gossip can also cause certain negative organizational feelings and behaviors (jealousy, exclusion, distrust, etc.) and are thus advised to approach the matter with caution. These results signal that organizational communication strategies should be developed by considering contextual conditions and actors’ perceptions, beyond being based solely on positive messages.

From a broader organizational standpoint, when open participation, inclusive recognition mechanisms, and a psychologically safe environment are established, the phenomenon of positive gossip can become a methodically organized and collectively embraced strategic effort. Furthermore, to ensure that positive gossip spreads constructively and ethically, training sessions or workshops on effective communication and feedback mechanisms can also be initiated.

In conclusion, while positive gossip has the potential to bring significant benefits to organizations, it should be treated as a complex phenomenon that requires careful attention and a strategic approach.

## Data Availability

The original contributions presented in the study are included in the article/Supplementary material, further inquiries can be directed to the corresponding author.
