# Supplementary material for: Beyond expectations: exploring the multifaceted role of positive gossip in organizations
Source: Front Psychol. 2026 Sep 18;17:1824097. doi: 10.3389/fpsyg.2026.1824097 (PMC13630514; doi:10.3389/fpsyg.2026.1824097)
Supplement: Supplementary file 1 [file Table_1.docx]

***Supplementary Material***

**1	Appendices**

**Appendix A. Interview Questions**

| **Question No.** | **Interview Question** | **Purpose / Focus** |
| --- | --- | --- |
| 1 | Have you witnessed the existence of positive gossip in your organization? | Identify awareness and recognition of positive gossip phenomena. |
| 2 | Do you engage in positive gossip? Why? | Explore personal engagement and motivations for participating in positive gossip. |
| 3 | In your opinion, is the content of the positive gossip work-related or non-work-related? | Distinguish between professional and non-professional contexts of positive gossip. |
| 4 | Among whom and about whom is positive gossip done? | Identify the main actors and targets involved in positive gossip. |
| 5 | Why do you think positive gossip is done in the organization? | Reveal perceived organizational and interpersonal motives for engaging in positive gossip. |
| 6 | What are the effects of positive gossip on your organization and its employees? | Understand perceived outcomes and impacts on individuals and the organization. |
| 7 | When and where (inside or outside the organization) is positive gossip done more frequently? | Identify spatial and temporal dynamics of positive gossip practices. |
| 8 | About which topics do you think positive gossip is done more frequently? | Explore recurring topics and themes in positive gossip conversations. |
| 9 | Do you think positive gossip develops spontaneously or is it done intentionally within your organization? | Assess spontaneity versus intentionality in positive gossip formation. |
| 10 | Does positive gossip occur in face-to-face environments or is it also done in virtual environments? | Examine the communication channels through which positive gossip occurs. |
| 11 | Do you think there is an expectation among those who engage in positive gossip that the conversations will remain confidential? | Explore confidentiality expectations and social norms regarding positive gossip. |
| 12 | Do you think positive gossip has advantages for the organization and its employees? | Highlight perceived positive effects of positive gossip on morale and cohesion. |
| 13 | Does positive gossip have disadvantages? | Identify potential negative implications or risks associated with positive gossip. |
| 14 | Do you think the spread of positive gossip within the organization should be managed? If yes, how? | Examine managerial perspectives on regulating or facilitating positive gossip. |

**Appendix B. Summary of Prior Research on Positive Organizational Gossip**

| **Study** | **Focus** | **Method** | **Key Findings** | **Research Gap Addressed by Study** |
| --- | --- | --- | --- | --- |
| **Zoromba, M. A. et al. (2024)** | Mediating role of psychological detachment between patient mistreatment and nurses’ caring behaviors; moderating role of supervisor positive gossip. | Quantitative; Cross-sectional; 341 Egyptian nurses. | Patient mistreatment decreases caring; supervisor PWG buffers this effect. | Few nursing studies explore gossip as boundary condition; cross-sectional limits causality. |
| **Shan et al. (2024)** | Positive vs. negative gossip’s effects on work engagement via OBSE and affective rumination. | Quantitative; Three-stage SEM; 302 Chinese employees. | Positive gossip increases engagement; negative gossip decreases it. | Lack of integrated dual-path models considering both valences. |
| **Zong, B., Zhang, L., Chu, X., & Qu, J. (2021)** | How positive workplace gossip (PWG) supports newcomer socialization via informational and friendship ties. | Quantitative survey; 280 participants from 10 SMEs in China. | PWG improves newcomer socialization; both tie types mediate the effect. | Prior focus mostly on negative gossip; need to address positive aspects and network effects. |
| **Spoelma, T. M. & Hetrick, A. L. (2021)** | Effect of team gossip on team performance via social loafing. | Quantitative; Three-wave survey with teams. | Positive gossip reduces loafing; negative gossip increases it. | Prior work viewed gossip only negatively; lab-based samples limit external validity. |
| **Ugwu, F. O. et al. (2022)** | Effect of customer incivility on work engagement; moderated by supervisor PWG and friendship. | Quantitative; Time-lagged survey; 258 Nigerian employees. | Customer incivility reduces engagement; PWG and friendships buffer this effect. | Western/Asian bias in literature; positive gossip underexplored. |
| **Wang, D. et al. (2022)** | PWG and job satisfaction; mediation by job insecurity and organizational identity. | Quantitative; SEM; 1,267 Chinese employees. | PWG boosts satisfaction via reduced insecurity and stronger identity. | Most studies ignore positive gossip; limited cultural scope. |
| **Dai, Y. et al. (2022)** | PWG and innovative behavior; mediated by loyalty, moderated by trust. | Quantitative; Two-wave survey; 327 Chinese employees. | PWG increases innovation via loyalty; stronger when trust is high. | Neglect of PWG’s role in innovation; limited mediators studied. |
| **Liao, G., Wang, Q., & Li, Y. (2022)** | How PWG reduces employee silence via psychological safety and promotion-focused regulation. | Quantitative; Two-wave time-lagged SEM; 311 Chinese employees. | PWG reduces silence; psychological safety mediates; promotion focus moderates. | Limited to Chinese innovative firms; self-report bias. |
| **Greenslade-Yeats, J. et al. (2023)** | Recipient’s interpretations of gossipers’ intent and relational effects. | Qualitative; 20 interviews; template analysis. | Intent seen as Genuine, Prosocial, or Self-serving; affects relationships differently. | Recipient’s perspective neglected in past research. |
| **Jiang, L. et al. (2019)** | Job insecurity’s effects on workplace friendship; moderating role of gossip tendency. | Quantitative; Cross-sectional; 286 Mturk workers. | Job insecurity lowers friendships; positive gossip buffers feature insecurity. | Neglect of interpersonal effects of job insecurity; causality unclear. |
| **Tassiello, V. et al. (2018)** | What kind of gossip is most shared (positive/negative, malicious/non-malicious). | Quantitative; Experimental (MTurk). | Positive, non-malicious gossip most shared; more so for in-group targets. | Research oversimplifies gossip polarity; lacks nuance on maliciousness. |
| **Kuo, C.-C. et al. (2018)** | Supervisor gossip’s role in LMX and ostracism. | Quantitative; Dyadic SEM; 218 Taiwan dyads. | Positive gossip improves LMX, which reduces ostracism. | Lacks attention to power dynamics; limited to high-power-distance culture. |
| **Grosser, T. J., Lopez-Kidwell, V., Labianca, G. & Ellwardt, L. (2012)** | Managerial understanding of gossip’s functions in organizations. | Conceptual/Managerial synthesis. | Gossip has both positive and negative outcomes; managers can use gossip channels constructively. | Synthesizes theory into practice; lacks empirical data. |
| **Sommerfeld, R. D. et al. (2008)** | Effect of multiple gossip statements on trust in reciprocity games. | Quantitative; Experimental design. | Multiple gossip statements maintain trust; sender decisions align with average ratings. | Artificial environment; lacks incentive for deception. |
| **McAndrew, F. T. et al. (2007)** | Evolutionary psychology of gossip as a strategy for status and control. | Quantitative; Experimental (scenario ranking). | Negative gossip about rivals spreads more; positive gossip about allies. | Needs replication in non-Western samples. |
| **Grosser, T. J., Lopez-Kidwell, V., & Labianca, G. (2010)** | Role of positive and negative gossip in organizations and how it diffuses through friendship vs. workflow ties. | Quantitative; Social Network Analysis; Semi-structured interviews; MRQAP; OLS regression. | Positive gossip relates to both friendship and workflow ties; negative gossip only to friendship ties. | Cross-sectional design limits causality; lacks contextual detail; small sample. |

**Appendix C: Table of statements, codes, categories and themes**

| Significant Statement | Code | Category | Theme |
| --- | --- | --- | --- |
| “: ...usually related to work. It (positive gossip) really improves the person and can also change the perspective of the people around you or show them the things they are not aware of. ” | Sharing information about the work itself | Communication | ***Contextual Content and Motives*** (Context-specific antecedents and motives) |
| “It (positive gossip) can be about people's personalities. In addition, work life and work-related issues are usually included in positive gossip.“ | Sharing information about personalities |  |  |
| “… it (positive gossip) is more about the way people do their job; that is, people are already revealing their characters while working, in fact, it is basically about the character of the person.“  “…it was also found that talking about the achievements of other members through "gossip" emerged as a motivating factor among the participants interviewed.“ | Sharing information about performance | Motivation |  |
| “When I see the achievements of successful people, I like to talk about it everywhere because I feel like I have achieved it.”  “I want the work to be seen and heard, that belongs to my friends. I wish the same could be done for me.” | Evaluative talk |  |  |
| “As I said before, people gossip positively with malicious intentions… If we do not like a person and because it is not possible to say that he/she achieved such success at work and that he/she did not deserve to get it, the easiest way to show these feelings is to mask them. Through using round sentences like “Did you see that he/she has applied to associate professorship, I hope it will be good for him/her and pass the subject.” | Emotion management through gossip | Strategy |  |
| “There is this thing in our society about positive gossip: people perceive saying nice things about each other as a part of a long-term plan. He/she's probably gossiping to look good for her. There may even be people who think that their kind words can reach that person... But if someone says something positive, I guess there might be something in it, there might be a closeness or there might be a relationship of interest with the person they are talking about.” | Self-serving motives in gossip |  |  |
| “There is no expectation for positive gossip to be kept secret.”  “I think not, I mean, there may be such expectations in negative gossip, but I think not in positive gossip” | Non-malevolent information exchange | Expectation of confidentiality | ***Social Boundaries and Conditional Privacy*** (Relational boundaries and interpersonal dynamics) |
| “It happens among the people who come into contact with each other during the day.”  “More like our close friends and the people you are trying to communicate with one-on-one”  “Usually with close friends again”  “Usually our colleagues, close friends especially” | Relational closeness |  |  |
| “I think they are not particularly talked about”  “No, it is not usually done consciously. You know, you open up spontaneously, finish the whole thing thoroughly. “  “No, it develops spontaneously. We don't say let's talk about this and that, it becomes an agenda at that day...” | Spontaneous gossip | Gossip initiation process | ***Emergence and Circulation*** (Emergence processes and interactional dynamics) |
| “Well, if there is no personal expediency then it is spontaneous. But if he/she has an interest, he brings it there and makes a positive statement. I would say flattery.”  “When the gossip is positive, sometimes it has a background…The gossiper usually comes with a request. Let's say her/his friend has a problem and the gossiper wants to find a solution. She/he goes to the person who has power to offer a solution and puts a good word about the friend in need… So, I think it's more likely to come with a premeditated request rather than spontaneous.” | Intentional gossip |  |  |
| “… conversations (positive gossip) about the work environment seem to be more common at work. I don't know, we go to the cafeteria, and we talk (positive gossip) while eating.”  “It is done inside the institution.”  “During daily working hours at the institution...”  “It is predominantly face-to-face…”  “It happens on social media too, but it doesn’t feel right to chat about third parties there.  “WhatsApp is a part of our lives for communication, but mainly face to face…” | Contextual environment of gossip | Processual unfolding of gossip |  |
| “In the institution, it is usually done when people finish their work and go to sit for tea with each other, and usually by closing the doors… but sometimes it also happens that when you talk to people you come together with outside of work, it is also opened, so it is also mentioned there.… “  “When we take a coffee break, then (positive) gossip takes place.”  “In school, especially during lunchtime, let me say that all the time (again, this is my opinion), so people are making time for it.” | Timing of gossip |  |  |
| “By the way, we work here with 20 people, 20 different characters, 20 different thoughts. If we perceive these people with their differences and then evaluate them with these differences, we cannot be united. We must surely find common ground. That's why I think it (positive gossip) brings people together outside of these differences and keeps the institution together.” | Gossip as a bridging mechanism | Building intra-organizational social bonds through gossip | ***Ambivalent Effects*** (Multilevel consequences for individuals and organizations) |
| “Well, I believe it strengthens the bonds. For example, I heard that my friend was thinking and worrying about my problem and talking about it when I wasn’t there. That's when I feel more like I belong there. Well, in a friend group or at work… knowing that you are being thought of, knowing that you are well spoken creates a positive effect at that moment.”  “Positive gossip strengthens people's communication.” | Bringing people closer together |  |  |
| “for example, ‘Did you hear that so-and-so applied to be an associate professor, how nice, she/he will become an associate professor in a short time’. These discourses seem to be positive, but I think there is a little bit of jealousy or envy behind them.” | Latent envy | Unintended relational disruption resulting from gossip |  |
| “Unfortunately, when the positive gossip is made among the colleagues, we have too many of them who want to abuse it or turn this current pleasant atmosphere in different directions with different and incomplete information.... After a certain time, positive gossip begins to turn against that colleague (about whom positive gossip is made). Because, unfortunately, people cannot accept this because they cannot digest it.” |  |  |  |
| “ …I think it has destructive effects in the organization when positive gossip takes place at the management level about the same person all the time, because none of us like it.”  “We do not always like someone to be praised positively, as if good things have always been gathered in a person's life and we do not have them. Because it reminds us of our own shortcomings, and we do not like to accept our imperfections as human beings“ | Perceived favoritism arising from gossip dynamics |  |  |

**Appendix D. Table of Participant Profile**

| **Organizational context** | **Role/position** | **Total (n)** | **Male** | **Female** |
| --- | --- | --- | --- | --- |
| Public agency | Employees | 12 | 8 | 4 |
| Academic institution | Academic staff | 14 | 5 | 9 |
| **Total** |  | **26** | **13** | **13** |
